# Supplementary material for: Frequency enrichment of coding variants in a French-Canadian founder population and its implication for inflammatory bowel diseases
Source: medRxiv. 2025 Jul 14:2025.07.11.25331388. Preprint. [Version 1] doi: 10.1101/2025.07.11.25331388 (PMC12338899; doi:10.1101/2025.07.11.25331388)
Supplement: Supplement 1 [file media-1.pdf]

Supplementary Material

Supplementary Figures

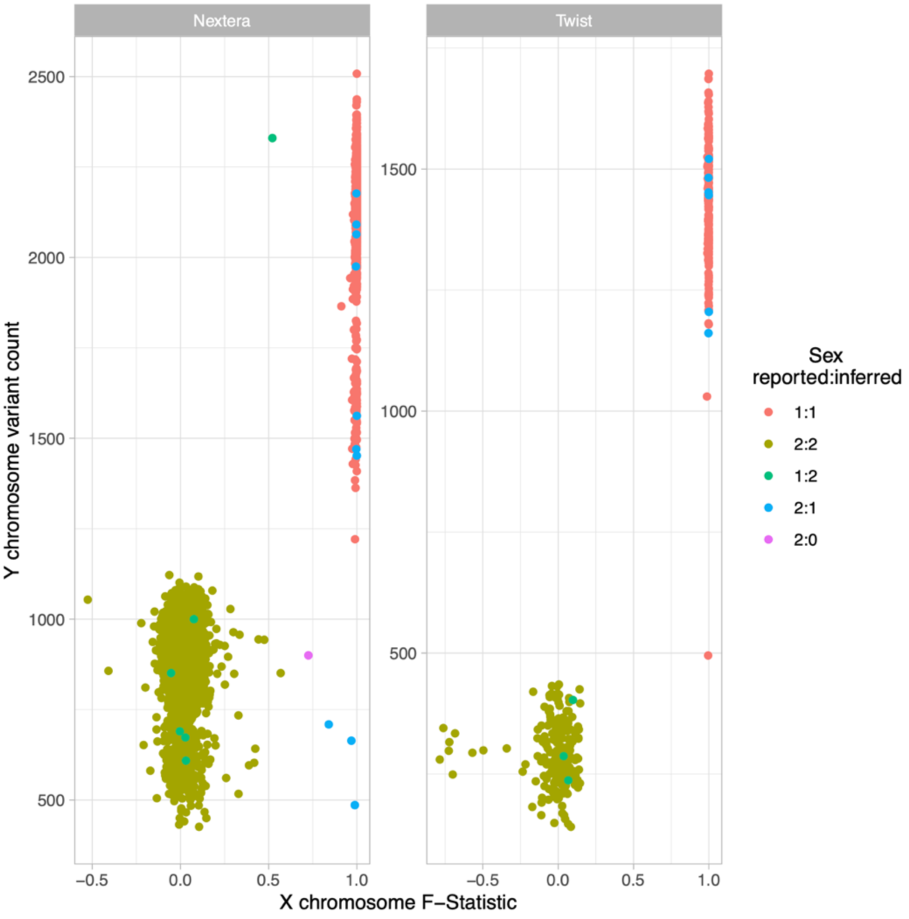

Supplementary Figure 1. Genetic sex inference. The X chromosome F-statistic was obtained with PLINK v1.9 using the --impute-sex 0.7 0.8 and --split-x options on a LD pruned dataset (--indep-pairwise 50 5 0.5) of common variants (MAF> 0.01) allowing for 10% missing rate. The number of non-missing Y chromosome variants were counted for each sample. Sex inference was performed separately for samples sequenced with Nextera and Twist captures (right and left panels, respectively). Each point represents one sample coloured according to sex reported in the metadata file (first number in the legend) versus sex inferred from genetic data (second number), where 1 refers to male, 2 to female and 0 to undetermined.

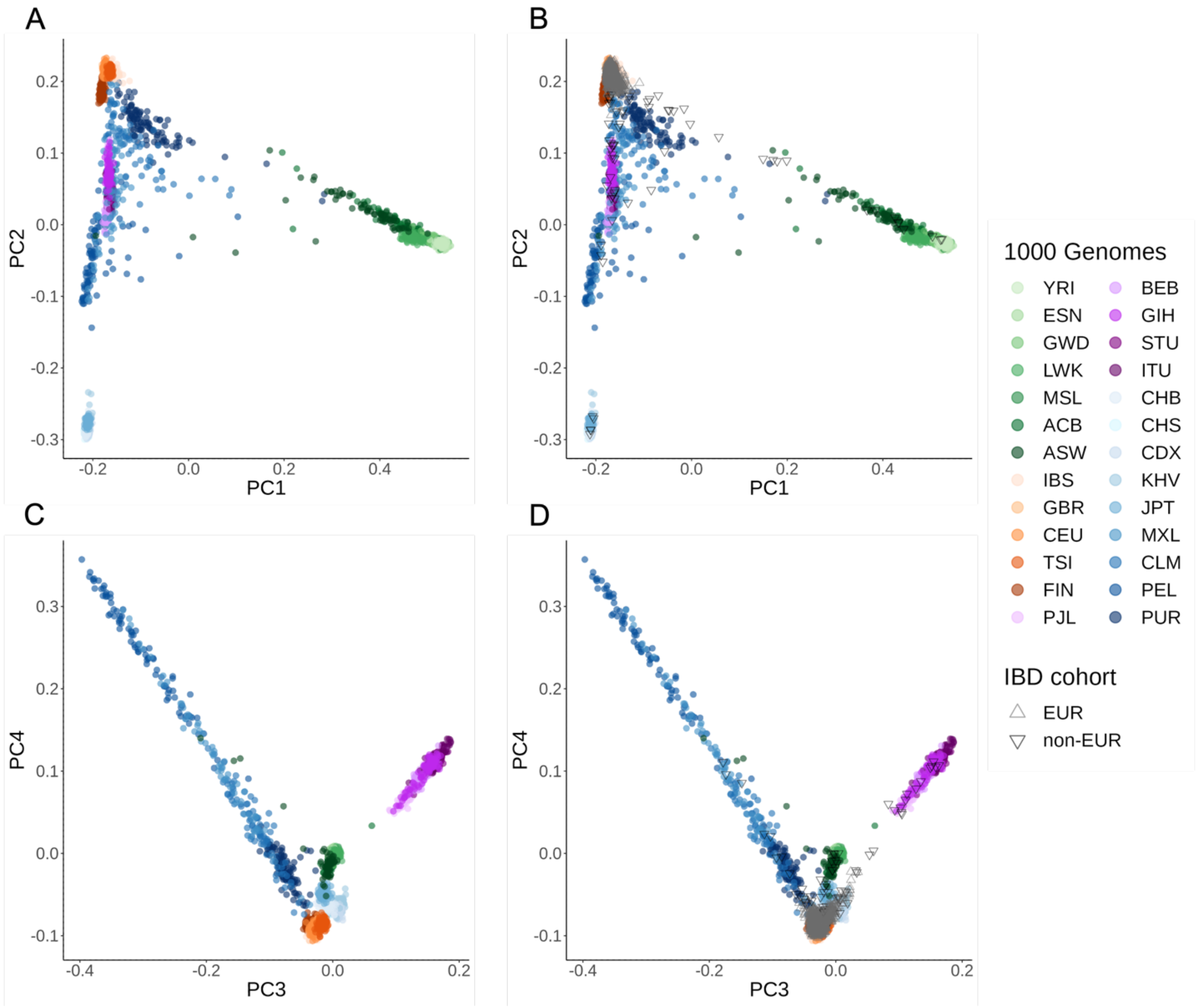

Supplementary Figure 2. Principal component analysis (PCA) analysis of worldwide populations. PCA of the 1000 Genomes and PCA projection of 3,015 samples from the Quebec IBD cohort. Upper panels (A-B) show PC1 vs PC2 and lower panels (C-D) PC3 vs PC4. The 1000 Genomes samples are represented by points colored according to 1000 Genomes superpopulation labels (AFR=green; EUR=orange; SAS=purple; AMR=blue). Sample abbreviations for the 1000 Genomes project are listed in Supplementary Table 3. Individuals from the Quebec IBD cohort (IBD cohort) are represented by up and down triangle symbols, respectively for individuals inferred most genetically similar to European (EUR) and non-European (non-EUR) samples from the 1000 Genomes project.

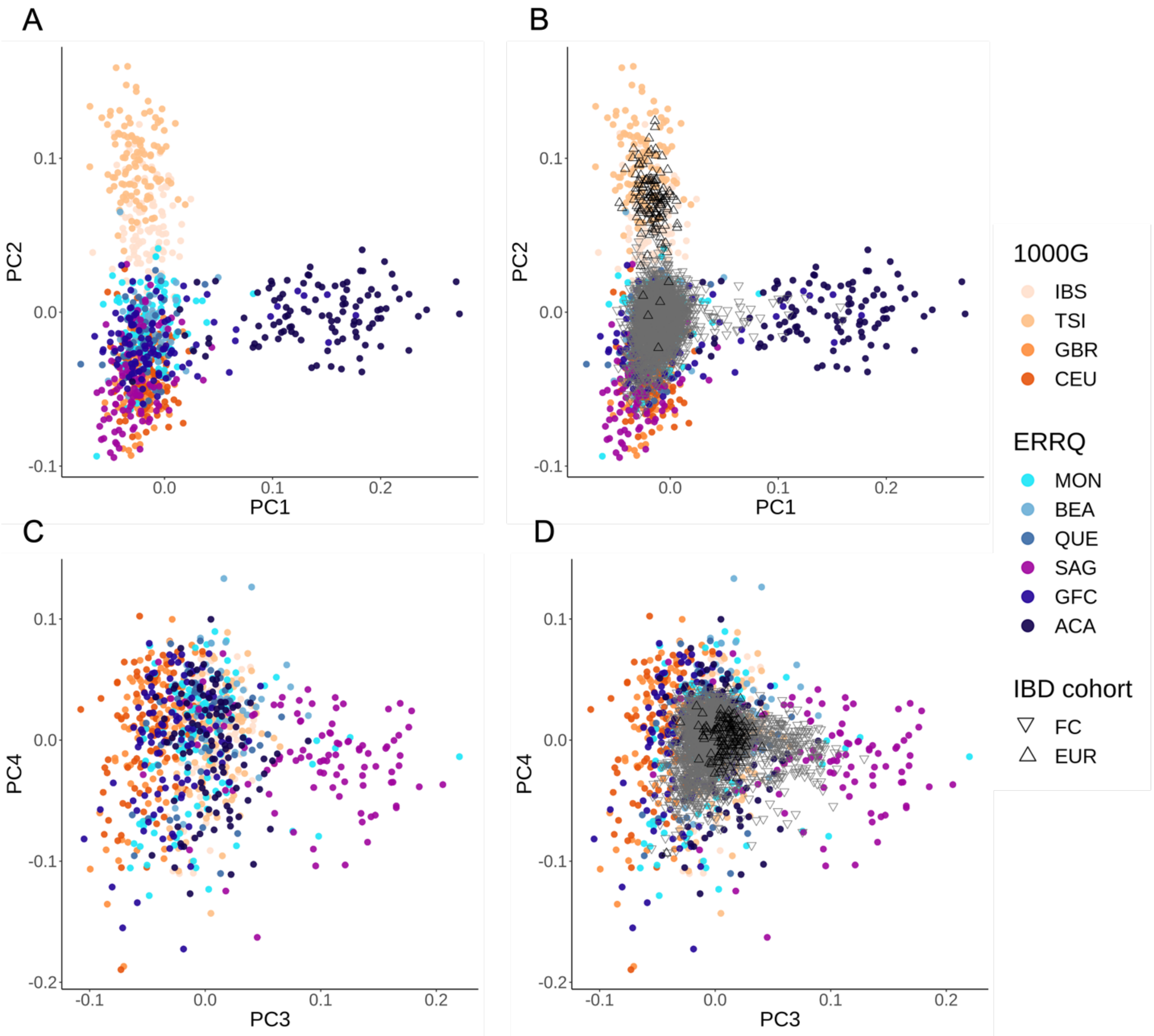

Supplementary Figure 3. PCA of samples with European ancestries. PCA of European population samples from the 1000 Genomes and Quebec samples from the ERRQ reference panel and PCA projection of IBD cohort samples. PCA is performed on a combined set of 4 European populations from the 1000 Genomes (excluding the Finnish sample) and a subset of the ERRQ. Upper panels show (A-B) PC1 vs PC2 and lower panels (C-D) PC3 vs PC4, with their respective proportion of variance explained (in %). Individuals from the IBD cohort are represented by down and up triangle symbols, respectively for individuals inferred most genetically similar to French Canadians (FC) from the ERRQ reference panel and to Europeans (EUR) population samples from the 1000 Genomes. Sample abbreviations for the 1000 Genomes project are listed in Supplementary Table 3, and those for the ERRQ in Supplementary Table 4.

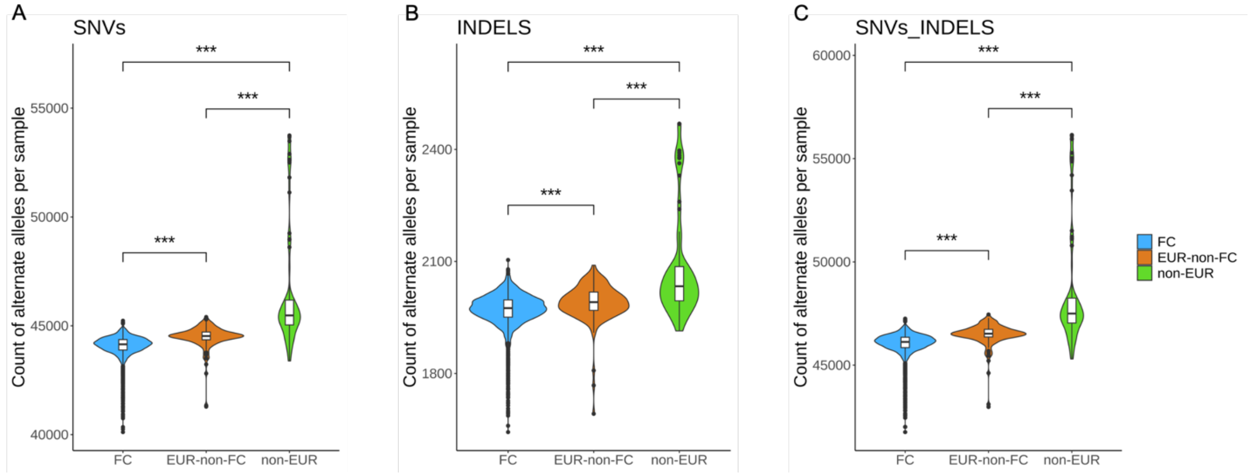

Supplementary Figure 4. Distribution of variants in three subsets of the Quebec IBD cohort. Violin plots represent the distribution of alternate alleles in three subsets of the Quebec IBD cohort, namely the FC subset, the EUR-non-FC and non-EUR subsets. Boxplots represent the median of the distribution and range between the first quartile (Q1, 25th percentile) and the third quartile (Q3, 75th percentile) and outliers are defined as datapoints outside of the smallest and largest values within the range defined as 1.5 times the IQR from the quartiles. Distributions were compared with a pairwise Wilcoxon test. All comparisons were significant (p-value < 0.001).

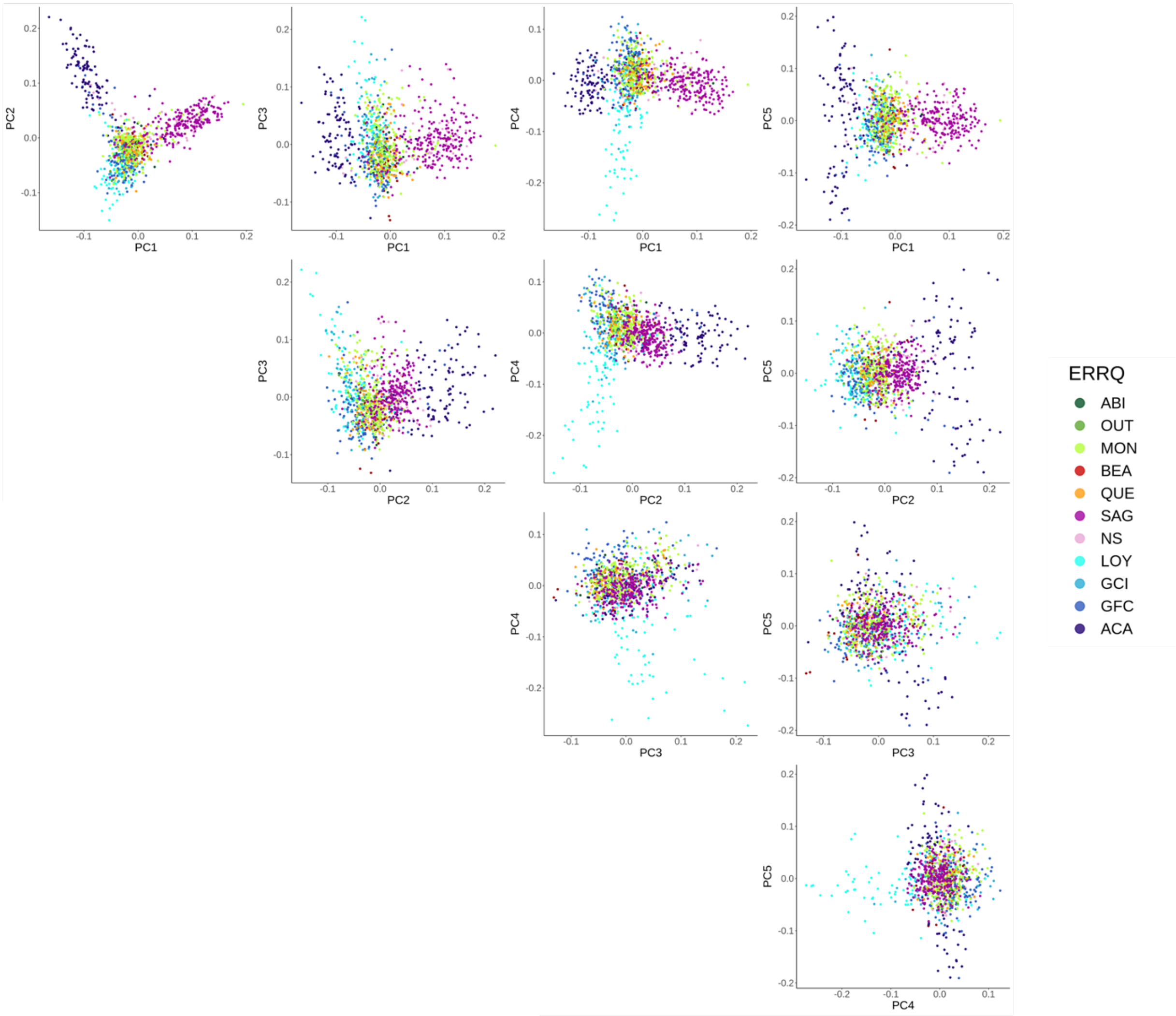

Supplementary Figure 5. Fine-scale structure of French Canadians using the ERRQ reference panel. PCA of the ERRQ reference panel. PC1 against PC5 are shown. Abbreviations: ABI: Abitibi-Témiscamingue; OUT: Outaouais; MON: Montreal; BEA: Beauce; QUE: Quebec City; SAG: Saguenay–Lac-Saint-Jean; NS: North-Shore; LOY: Gaspesia Loyalist; GCI: Gaspesia Channel Islanders; GFC: Gaspesia French Canadians; ACA: Gaspesia Acadians.

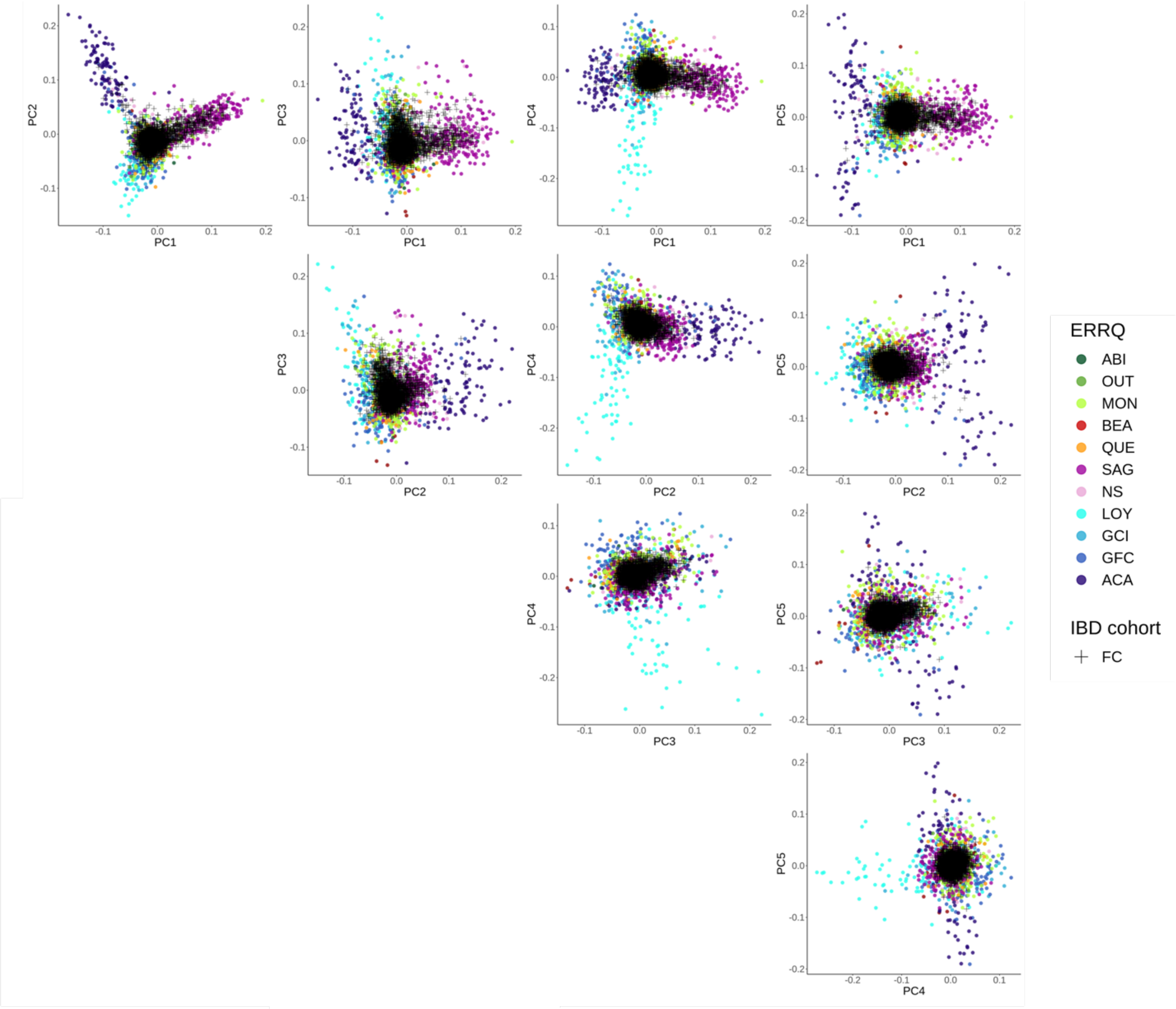

Supplementary Figure 6. Fine-scale structure of French Canadians using the ERRQ reference panel and projection of the FC subset. PCA of the ERRQ reference panel and projection of the FCIBD subset. PC1 against PC5 are shown. Abbreviations: ABI: Abitibi-Témiscamingue; OUT: Outaouais; MON: Montreal; BEA: Beauce; QUE: Quebec City; SAG: Saguenay–Lac-Saint-Jean; NS: North-Shore; LOY: Gaspesia Loyalist; GCI: Gaspesia Channel Islanders; GFC: Gaspesia French Canadians; ACA: Gaspesia Acadians, FC: French-Canadians

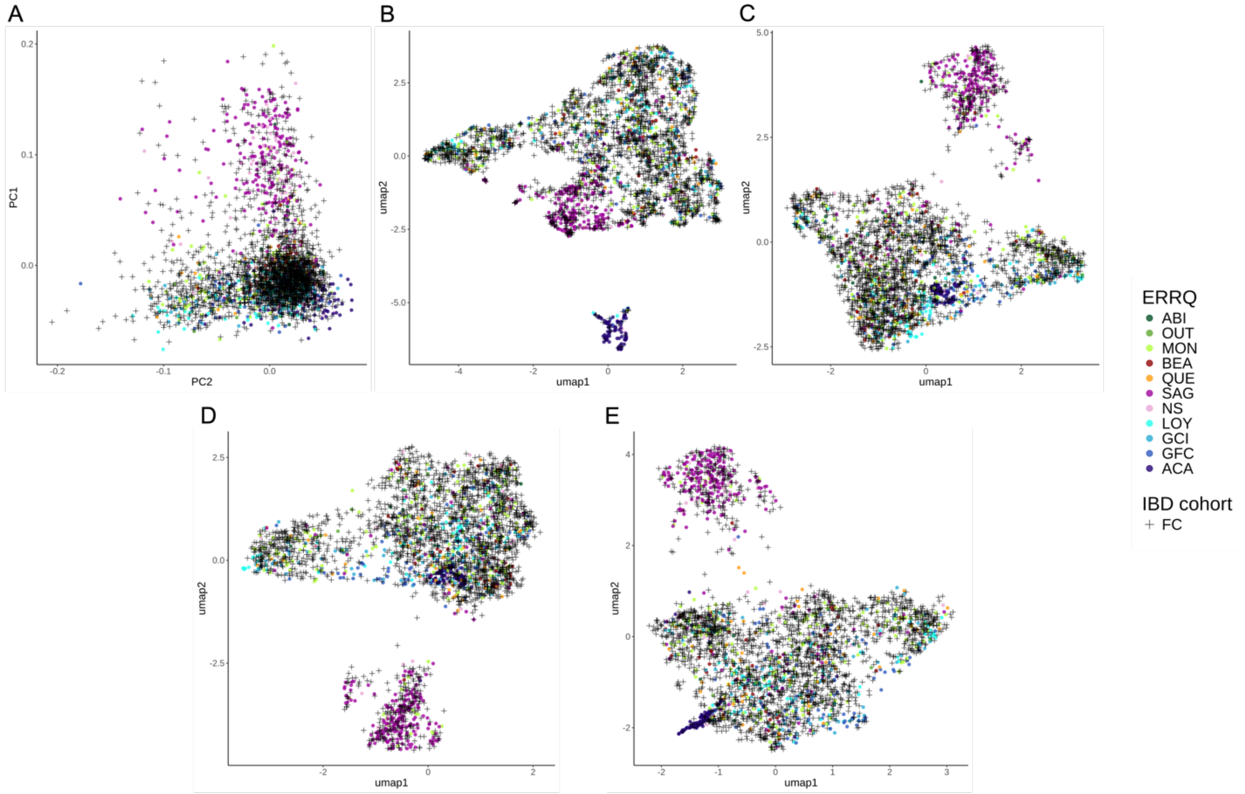

Supplementary Figure 7. **Uniform Manifold Approximation and Projection (UMAP) of the ERRQ and UNRFC samples.** (A) Principal component analysis representing PC1 and PC2. B) UMAP of the top 5 PCs. (C) UMAP of the top 10 PCs. D) UMAP of the top 15 PCs. E) UMAP of the top 20 PCs. Abbreviations: ABI: Abitibi-Témiscamingue; OUT: Outaouais; MON: Montreal; BEA: Beauce; QUE: Quebec City; SAG: Saguenay–Lac-Saint-Jean; NS: North-Shore; LOY: Gaspesia Loyalist; GCI: Gaspesia Channel Islanders; GFC: Gaspesia French Canadians; ACA: Gaspesia Acadians, FC: French-Canadians

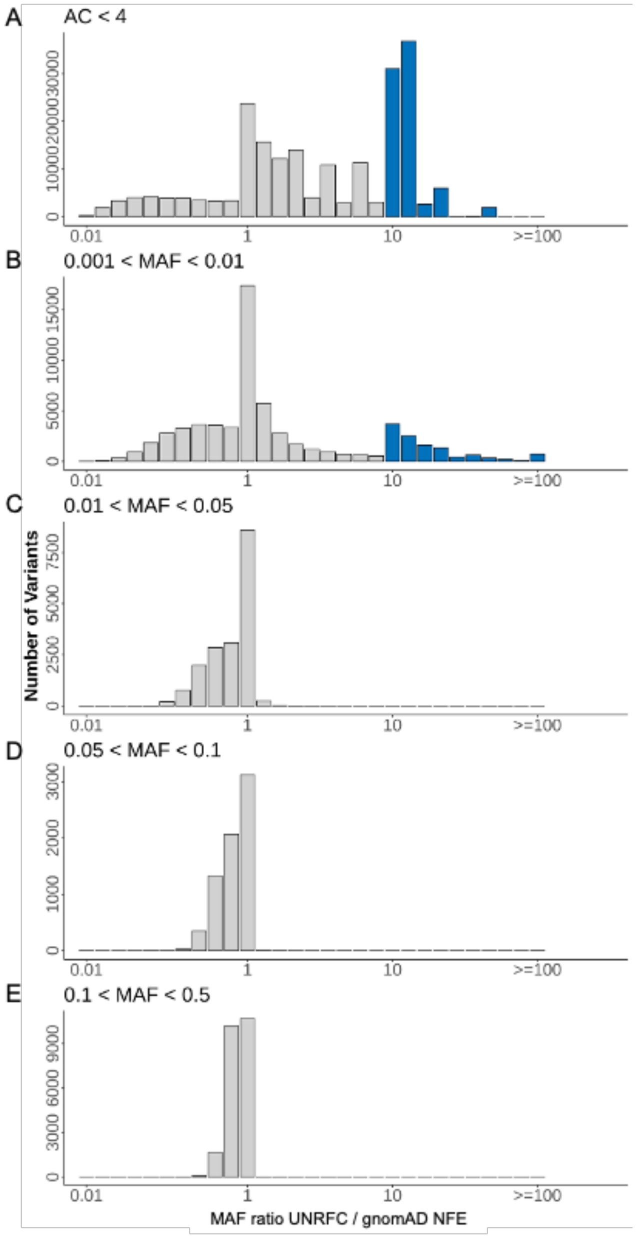

Supplementary Figure 8. Enrichment of protein-coding variants per frequency categories. Histograms show the distribution of variants as a function of their minor allele frequency (MAF) ratio in the UNRFC sample relative to gnomAD NFE exomes. Variants with MAF ratio equal or above 10-fold enrichment in the UNRFC are highlighted in blue. Frequency enrichment histograms are shown for variants in different MAF categories in the UNRFC subset, specifically (A) allele count (AC) < 4 (MAF = 0.001), (B) 0.001 < MAF < 0.01, (C) 0.01 < MAF < 0.05, (D) 0.05 < MAF < 0.1, (E) 0.1 < MAF < 0.5.

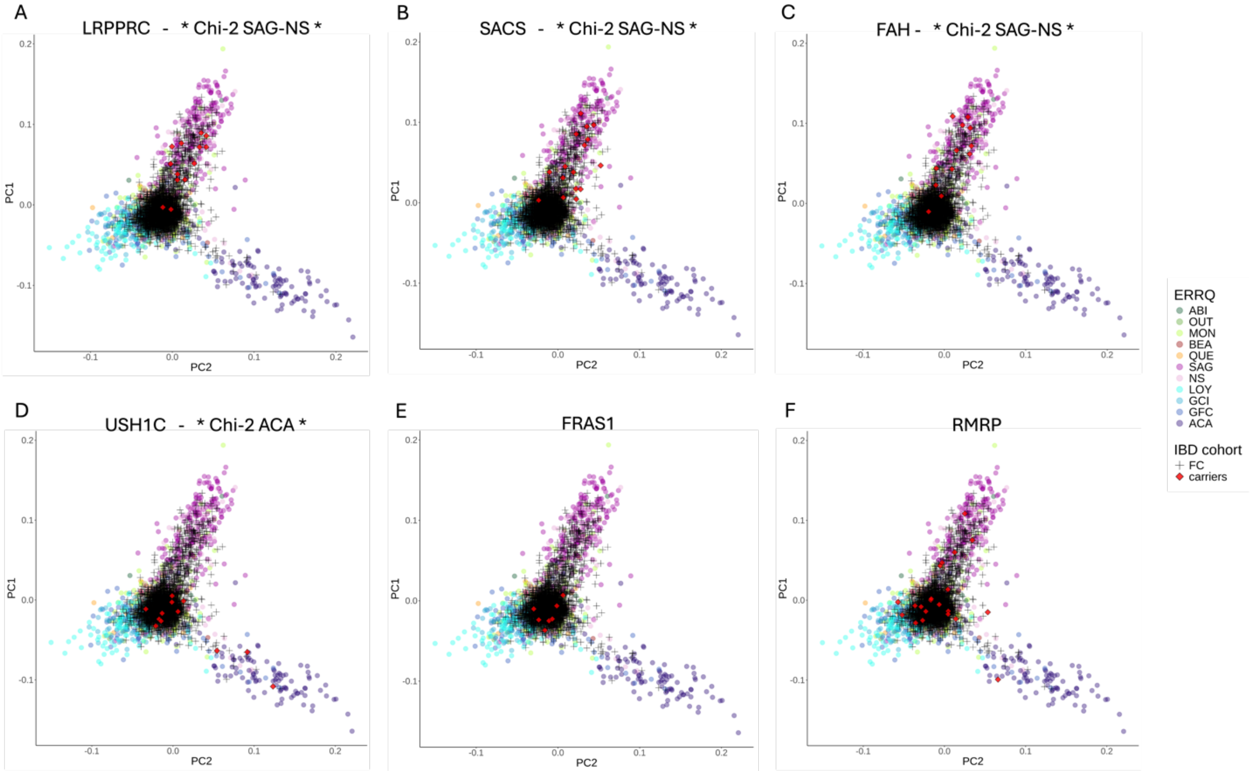

Supplementary Figure 9. PCA plots highlight carriers of PLP variants in the UNRFC subset. The PCA was performed on the ERRQ reference panel, and the exomes from the UNRFC subset were then projected on this PCA. Carriers of PLP variants are highlighted in red. Abbreviations: ABI: Abitibi-Témiscamingue; OUT: Outaouais; MON: Montreal; BEA: Beauce; QUE: Quebec City; SAG: Saguenay–Lac-Saint-Jean; NS: North-Shore; LOY: Gaspesia Loyalist; GCI: Gaspesia Channel Islanders; GFC: Gaspesia French Canadians; ACA: Gaspesia Acadians, FC: French-Canadians.

Supplementary Tables

Supplementary Table 1. Summary of the Quebec IBD cohort by study cohort before and after quality control (QC). Study cohorts are described in Materials and Methods and include IBDGC-Montreal, iGenomed-MTT, Genome Quebec-GENIZON Case-Control (GC_CC), Genome Quebec-GENIZON Trios (GC_Trios).

Study cohort	Before QC	After QC
IBDGC-Montreal	995	931
IGenomed-MTT	186	169
GC_CC	872	869
GC_Trios	1,049	1,046
Total	3,102	3,015

Supplementary Table 2. Summary of quality control steps and filtering.

	Number of samples	Number of variants	Genotyping rate	Number of sites removed
**Initial variant callset**	**3,102**	**4,435,411**	**0.6784**	
**Genotype-level filters**				
Genotype quality > 20	3,102	4,435,411	0.5890	-
PASS	3,102	3,826,429	0.5816	608,982
Allele balance ratio	3,102	3,826,429	0.5793	-
Normalize InDels	3,102	3,826,429	0.5793	-
**Individual-level filters**				
No Duplicated Samples	3,074	3,826,429	0.5794	-
No Sex Mismatch, chimera, contamination	3,015	3,826,429	0.5805	-
**Genotype-/variant- level filters**				
**Autosomes**	**3,015**	**3,729,594**	**0.5806**	
DP > 10	3,015	3,729,594	0.5466	-
Mendelian errors set as missing	3,015	3,729,594	0.5466	-
Missing < 5%	3,015	1,258,779	0.9928	2,470,815
Hardy Weinberg p-value < 1 x 10-6	3,015	1,258,415	0.9928	364
Matching exome kits with 50bp flanks	3,015	848,032	0.9956	410,383
**Total after quality control filters**	3,015	848,032	0.9956	-

Supplementary Table 3. 1000 Genomes cohort populations description. The superpopulation labels stand for: AFR = Africa, AMR = America, EAS = East Asia, EUR = Europe, SAS = South Asia.

Sample abbreviation	Superpopulation	Description
ACB	AFR	African Caribbean in Barbados
ASW	AFR	African Ancestry in Southwest US
BEB	SAS	Bengali in Bangladesh
CDX	EAS	Chinese Dai in Xishuangbanna, China
CEU	EUR	Utah residents with Northern and Western European ancestry
CHB	EAS	Han Chinese in Bejing, China
CHS	EAS	Southern Han Chinese, China
CLM	AMR	Colombian in Medellin, Colombia
ESN	AFR	Esan in Nigeria
FIN	EUR	Finnish in Finland
GBR	EUR	British in England and Scotland
GIH	SAS	Gujarati Indian in Houston,TX
GWD	AFR	Gambian in Western Division, The Gambia
IBS	EUR	Iberian populations in Spain
ITU	SAS	Indian Telugu in the UK
JPT	EAS	Japanese in Tokyo, Japan
KHV	EAS	Kinh in Ho Chi Minh City, Vietnam
LWK	AFR	Luhya in Webuye, Kenya
MSL	AFR	Mende in Sierra Leone
MXL	AMR	Mexican Ancestry in Los Angeles, California
PEL	AMR	Peruvian in Lima, Peru
PJL	SAS	Punjabi in Lahore,Pakistan
PUR	AMR	Puerto Rican in Puerto Rico
STU	SAS	Sri Lankan Tamil in the UK
TSI	EUR	Toscani in Italy
YRI	AFR	Yoruba in Ibadan, Nigeria

Supplementary Table 4. ERRQ cohort regional and ethno-cultural groups description.

Abbreviation	Regional or ethno-cultural population	Geographical Region in Quebec	Number of samples
ABI	Abitibi	North-West	24
OUT	Outaouais	West	17
MON	Montreal	Central	215
BEA	Beauce	Central	44
QUE	Quebec	Central	53
SAG	Saguenay–Lac-Saint-Jean	North-East	232
NS	North-Shore	North-East	71
LOY	Gaspesia Loyalist	East	94
GCI	Gaspesia Channel-Islanders	East	91
GFC	Gaspesia French-Canadian	East	106
ACA	Gaspesia Acadia	East	101

Supplementary Table 5. Detailed description of IBD phenotypes in the FC subset.

	Case/Control			Trios
	**IBD***	**CD**	**UC**	**CD index**
Total	1016	768	228	318
**Sex**				
Female	582	454	117	205
Male	434	314	111	113
**Age at diagnosis**				
Median [Q1-Q3]	27 [19-40]	27 [19-40]	28 [21-40]	21[17-26]
Missing	38	32	6	5
**Disease Location CD**				
Ileal (L1)	-	219	-	87
Colorectal (L2)	-	192	-	76
Ileocolonic (L3)	-	333	-	155
Missing	-	24	-	0
**Disease Behavior CD**				
Inflammatory (B1)	-	378	-	142
Stricturing (B2)	-	145	-	57
Penetrating (B3)	-	222	-	119
Missing	-	23	-	0
**Perianal CD**				
Yes	-	102	-	0
No	-	293	-	0
Missing	-	373	-	318
**Disease Extent UC**				
Proctitis (E1)	-	-	27	-
Left-Sided (E2)	-	-	104	-
Extensive (E3)	-	-	89	-
Missing	-	-	9	-

*Indeterminate Colitis (n=20) is included in the IBD subset with CD and UC cases

Supplementary Table 6. Variant summary for the Quebec IBD cohort. Number of variants, SNVs and InDels after quality controls. Novel variants are those not in dbSNP.

Variants	All (N)	All (% singletons)	Novel (N)	Novel (% singletons)	In dbSNP (N)	In dbSNP (% singletons)
All	848,032	40.96	52,994	58.98	795,038	39.76
SNV	804,034	41.23	46,082	62.21	757,952	39.96
InDel	43,998	36.00	6,912	37.44	37,086	35.73

Supplementary Table 7. Summary of exome data by subset of the Quebec IBD cohort. The FC subset contains individuals inferred most genetically similar to FC reference samples. These samples were included in single-point association test and burden test. The UNRFC subset contains individuals inferred most genetically similar to FC reference samples, unrelated up to the third degree. The UNRFC subset was used in enrichment analyses. The FC controls subset contains control-only individuals inferred most genetically similar to FC reference samples. This subset was used to compute enrichment statistics for variants identified in association tests. The EUR-non-FC subsets and non-EUR subsets were used to describe the distribution of coding variants per exomes.

	Number of samples	Number of variants		
		All	SNVs	InDels
All	3,015	848,032	804,034	43,998
FC	2,820	653,574	623,119	30,455
UNRFC	2,323	640,073	610,326	29,747
FC controls	760	369,924	352,747	17,177
EUR-non-FC	127	203,128	193,870	9,258
Non-EUR	68	227,573	217,357	10,216

Supplementary Table 8. Descriptive statistics of the Quebec IBD cohort, the FC and the UNRFC subsets.

**Demographic and clinical characteristics**	**All**	**FC**	**UNRFC**
Number of participants	3,015	2,820	2,323
Number of women (%)	1,726 (57.25)	1,615 (57.30)	1,321 (56.87)
Number of IBD patients	1,507	1,364	1,002
Number of parents	699	696	637
Number of controls	809	760	684
**IBD phenotypes of patients**			
Crohn's disease	1,207	1,116	769
Ulcerative colitis	275	228	214
Indeterminate colitis	25	20	19

Supplementary Table 9. Number of variants per exome in the FC, non-EUR, and EUR-non-FC subsets. The mean number of variants per exome is shown, as well as standard deviation (s.d.), variance, median and 5th and 95th percentile of the distribution.

Variant type	Population	mean	s.d.	variance	median	5th perc	95th perc
SNVs + InDels	FC	46,021.86	577.09	333,034.55	46,126.00	44,984.95	46,649.15
	non-EUR	48,536.69	2,858.40	8,170,426.01	47,499.00	46,067.00	55,189.95
	EUR-non-FC	46,450.68	601.31	361,575.68	46,523.00	45,549.40	47,121.50
	ALL	46,096.64	808.13	653,070.10	46,146.00	45,037.10	46,765.60
SNVs	FC	44,053.21	539.56	291,128.73	44,149.00	43,087.95	44,653.05
	non-EUR	46,456.59	2,721.68	7,407,523.29	45,477.00	44,109.40	52,805.30
	EUR-non-FC	44,462.22	560.82	314,515.25	44,529.00	43,564.90	45,105.70
	ALL	44,124.65	763.13	582,371.61	44,171.00	43,135.70	44,766.30
InDels	FC	1,968.64	49.55	2,454.84	1,975.00	1,882.00	2,030.00
	non-EUR	2,080.10	140.42	1,9716.66	2,033.50	1,949.40	2,388.55
	EUR-non-FC	1,988.46	50.99	2,600.35	1,991.00	1,936.20	2,054.00
	ALL	1,971.99	55.93	3,128.63	1,976.00	1,885.00	2,036.00

Supplementary Table 10. Variant summary of coding variants by consequence in the UNRFC subset.

Variants	All (N)	All (% singletons)	Novel (N)	Novel (% singletons)	In dbSNP (N)	In dbSNP (% singletons)
All	398,626	45.7	17,759	90.8	380,867	43.6
Synonymous	138,836	40.9	4,325	90.4	134,511	39.3
Non-synonymous	246,067	48	11,848	91	234,219	45.9
Splice	3,017	56.3	346	91.3	2,671	51.8
Frameshift	6,147	56.8	927	90.6	5,220	50.8
Inframe	4,526	46	309	88.3	4,217	42.9

Supplementary Tables in Excel File: SUPP_Data1_PLP_variants.xlsx

Supplementary Table 11. Previously described French Canadian founder variants. Curated list of known pathogenic variants for Mendelian diseases previously described with founder effect in French Canadians.

Supplementary Table 12. ClinVar PLP variants. Variant annotations for ClinVar PLP variants in the UNRFC unrelated subset.

Supplementary Table 13. ClinVar PLP variants in the *RMRP* gene in the UNRFC subset.

Variant (hg38) dbSNP	HGVSc	Consequence	ClinVar ID	ClinVar significance	AC	AN	NS	Carrier Rate
9:35657801:G:A rs1021056174	ENST00000363046.1 n.217C>T	Noncoding transcript exon variant	2445847	Variant of unknown significance - 1 star	1	4646	2323	1/2323
9:35657872:C:T rs753874439	ENST00000363046.1 n.146G>A	Noncoding transcript exon variant	379208	Pathogenic - 2 stars	1	4646	2323	1/2323
9:35657948:T:C rs199476103	ENST00000363046.1 n.70A>G	Noncoding transcript exon variant	14208	Pathogenic/Likely Pathogenic 2 stars	12	4646	2323	1/194
9:35658014:G:C rs772443941	ENST00000363046.1 n.4C>G	Noncoding transcript exon variant	552477	Pathogenic/Likely Pathogenic 2 stars	1	4624	2312	1/2312

Supplementary Table 14. Pathogenic variants observed in RMRP in 5 probands diagnosed with CHH in a tertiary care center.

VarID (hg38)	Preferred name	Consequence	ClinVar ID	ClinVar significance	AC	AN	AC (FC)	AN (FC)
9:35657948:T:C rs199476103	NR_003051.4(RMRP) n.72A>G	Noncoding transcript variant	14208	Pathogenic/Likely Pathogenic - 2 stars	6	10	6	8
9:35657807:G:C rs192060920	NR_003051.4(RMRP) n.213C>G	Noncoding transcript variant	632970	Pathogenic/Likely Pathogenic - 2 stars	2	10	0	8
9:35657804:T:A -	NC_000009.12 g.35657804T>A	Noncoding transcript variant	2078709	Variant of unknown significance - 1 star	1	10	1	8
9:35657825:C:T rs761398394	NR_003051.4(RMRP) n.195G>A	Noncoding transcript variant	928882	Pathogenic/Likely Pathogenic - 2 stars	1	10	1	8

Supplementary Tables in Excel Files : SUPP_Data2_FC_IBD_Association.xlsx

Supplementary Table 15. Association and enrichment results in known causal variants.
Supplementary Table 16. Novel association results.
Supplementary Table 17: SKAT-O Burden test results for the top 20 genes for non-synonymous and LoF variants.
Supplementary Table 18. Summary of key genes and function.
